# Advances in Biomarkers and Diagnostics in Periodontitis and Oral Diseases

**DOI:** 10.3390/ijerph18041886

**Published:** 2021-02-15

**Authors:** Gaetano Isola

**Affiliations:** Department of General Surgery and Surgical-Medical Specialties, School of Dentistry, University of Catania, Via S. Sofia 78, 95124 Catania, Italy; gaetano.isola@unit.it; Tel.: +39-095-378-2453

## 1. Introduction

Oral health is essential to general health and well-being at every stage of life [1]. A healthy mouth enables the nutrition of the physical body and enhances social interaction and promotes self-esteem and feelings of well-being [2]. The mouth serves as a “window” to the rest of the body, providing general health disorders signals. Oral and periodontal conditions have an impact on overall health and disease [3]. Bacteria from the mouth can cause infection in other parts of the body when the immune system has been compromised by disease or medical treatments (e.g., infective endocarditis) [4,5,6]. Systemic conditions and their treatment are also known to impact on oral health (e.g., reduced saliva flow, altered balance of oral microorganisms) [7,8].

The most important risk factors for oral cancer, the Oral Squamous Cell Carcinoma (OSCC) development, are tobacco use and extensive alcohol consumption. Moreover, infection with oncogenic types of human papillomavirus (HPV) has been identified as a significant risk factor for a subset of oral cancer [9,10].

The main reasons for failure in oral cancer treatment and the strongest adverse factors for prognosis are the spread to the regional lymph nodes and early development of local recurrence or second primary tumors [11]. Modern head and neck oncology can offer a plethora of treatment modalities including surgical resection with reconstructive options, radiotherapy (including proton therapy), conventional and targeted systemic treatment, and also, as of late, immunotherapy [12]. The success rates of treatments can significantly vary between particular patients. Thus, one of the biggest future challenges is to tailor multidisciplinary treatments such that they are based not only on clinical assessment of disease advancement determined by stage but also on the biological factors of the tumor.

Advances in genetics and molecular biology have improved our knowledge of cellular mechanisms, which provide insights into the pathophysiological processes that turn healthy epithelial cells into cancer. Potential biomarkers and therapeutic targets can be investigated to identify genetic signatures that could be used for early diagnosis, treatment personalization, and, finally, the prognosis for individual patients [13].

Numerous biomarkers are being utilized, including circulating tumor DNA (ctDNA), micro RNAs, extracellular vesicles, circulating tumor cells and endothelin receptor type B hypermethylation [14,15]. More than one-hundred salivary components have been reported to differ in concentration in patients with and without OSCC [16,17]. 

In this regard, saliva has been described as “the defender of the oral cavity” and provides for the protection of hard and soft tissues; aids taste, swallowing and digestion; and offers antimicrobial properties [18].

Saliva contains more than two thousand proteins, enzymes, electrolytes, small organic molecules, and antimicrobials. The whole saliva contains plasma-derived components, sloughed epithelial cells, microorganisms and their associated products, gingival crevicular fluid, debris, and nasopharyngeal discharge. In the context of oral disease, the research, identification and use of salivary biomarkers is ongoing for many conditions. In this editorial, we will discuss some of the salivary biomarkers researched and used for periodontal disease and oral/oropharyngeal cancer [19].

The aim of the special issue “New Biomarkers and Diagnostics in Oral Cancer and Oral Diseases” was to provide insight into the recent advances in the field of oral cancer and oral diseases. More specifically, among published manuscripts, Mazurek-Mochol et al. examined the association between the IL-17F rs763780 and IL-17A rs2275913 polymorphisms and periodontitis in non-smoking and smoking patients to check if these polymorphisms could be a risk factor for periodontitis. Interestingly, they found a lack of statistically significant associations between IL-17F rs763780 and IL-17A rs2275913 polymorphisms and periodontitis in a European population [20].

Recently, an increasing number of reports in literature have focused attention on some salivary research initiatives, the key objective of which is to generate a growing appreciation of the importance of saliva for overall health and for the diagnosis of oral diseases, and also to encourage a call to action in scientists and leaders in oral health to obtain the benefits of salivary screening tests useful for an advance detection of oral disease that will definitely help to stratify the patient’s risk and to reduce the global burden according to the “personalized medicine” approach [21,22,23,24]. It is ever more apparent that addressing this challenge to improve oral health worldwide will require a closer and more robust engagement across sectors in the dental field and the adoption of an upstream approach to reduce the global burden of disease in general.

This diagnostic modality in the field of molecular biology has led to the discovery and potential of salivary biomarkers for the detection of oral cancers. Biomarkers are the molecular signatures and indicators of normal biological, pathological process, and pharmacological response to treatment, and hence may provide useful information for detection, diagnosis, and prognosis of the disease. Saliva’s direct contact with oral cancer lesions makes it a more specific and potentially sensitive screening tool, whereas there are more than 100 salivary biomarkers. However, because of sensitivity and specificity, as well as technical requirements and cost, the use of salivary biomarkers is promising and has been confined to the laboratory. However, future investigations will continue along different pathways using different techniques to better understand salivary biomarkers’ role in oral health and disease.
